# Supplementary material for: Building confidence in quantitative systems pharmacology models: An engineer's guide to exploring the rationale in model design and development
Source: CPT Pharmacometrics Syst Pharmacol. 2017 Feb 9;6(3):156–67. doi: 10.1002/psp4.12157 (PMC5351409; doi:10.1002/psp4.12157)
Supplement: Supplementary file 1 — Supplementary Figure S1 Summary of immune response associated with granuloma formation in leishmaniasis. (a) Within hours of experimental infection with Leishmania donovani, dendritic cells present parasite antigens to naive T lymphocytes in lymphoid tissues to initiate an adaptive immune response. (b) Simultaneously, parasites in the liver infect resident liver macrophages (Kupffer cells), stimulating the production of chemokines that attracts innate lymphoid cells (of which NKT cells are best characterized). NKT cells engage with infected Kupffer cells via cognate receptor‐ligand interactions, amplifying the chemokine response to attract additional Kupffer cells, NKT cells, and eventually other cell types (see d, below). (c) Over the first few days of infection, T cells differentiate into a variety of subsets (Th1, Th2, Treg), producing cytokines that may cross‐inhibit or cross‐stimulate T‐cell differentiation. These cytokines also promote (e.g., interferon [IFN]) or inhibit (e.g., interleukin [IL‐10]) the ability of macrophages to kill Leishmania. (d) The relative balance of different T‐cell subsets, together with monocytes, dendritic cells, and occasionally neutrophils that are attracted to the expanding granuloma determines parasite burden. Notably, granuloma development is asynchronous (lower right). (e) Reduction in parasite burden is achieved when Th1‐type immune responses become dominant. (f) Resolution of infection is accompanied by granuloma involution (loss of cellularity) and a restoration of homeostasis. Experimental and modeling data suggest, however, that some residual parasites survive in some granulomas due to regulatory mechanisms. [file PSP4-6-156-s001.pdf]

Building Confidence in Quantitative Systems Pharmacology Models: An Engineer's Guide to Exploring the Rationale in Model Design and Development

J. Timmis[1,2,*,%], K. Alden[1,%],P. Andrews[2], E. Clark[2], A. Nellis[2], B. Naylor[1,2], M. Coles [3], P. Kaye [3]

[1]Department of Electronics, The University of York, UK.

[2]SimOmics Limited, 106 Heworth Green York UK.

[3]Centre for Immunology and Infection, Hull York Medical School/University of York, York. UK.

[*]Corresponding author: jon.timmis@york.ac.uk

[%]Authors contributed equally to this work

Word count introduction 1123

Word count body 5893

This tutorial promotes good practice for exploring the rationale of systems pharmacology models. A safety systems engineering inspired notation approach provides much needed rigour and transparency in development and

application of models for therapeutic discovery and design of intervention strategies. Structured arguments over a models development, underpinning biological knowledge, and analyses of model behaviours, are constructed to determine the confidence that a model is fit for the purpose for which it will be applied.

Introduction

When constructing a quantitative systems pharmacology (QSP) model there are many issues to consider, from what aspects of the biological system needs to be modelled, hence defining the scope of the model, to what modelling approach to use, through to how the model is developed, and what abstractions are to be made during the model development process. Likewise, there may be existing models that have been developed and are in use as part of an experimental study, but which may be seen as a blackbox where the rationale for their construction, use, and analysis is undocumented or was never coherently established.

During model development various decisions have to be made, such as the inclusion of simplifications and assumptions in place of biological knowledge, which may be very reasonable but often are forgotten about or poorly documented. Yet these decisions impact the relationship between any predictions that model generates and the real biological system the model is aiming to capture, in turn impacting the level of confidence a researcher has in applying those predictions within their own studies. Work in Alden et al [4] presented a tool, Artoo, that permits the application of an adapted version of Goal Structuring Notation (GSN) [30] through which a structured argument is developed to show that a model is fit for the purpose for which it has been conceived. Within the context of modelling, an argument is constructed by making claims concerning aspects of model development, which are, where possible, supported by available evidence. In their description, Alden et al provide an overview of using argumentation to examine fitness for purpose, exemplifying application of the approach to explore the rationale underlying the development of a previously published simulation of secondary lymphoid organ development [7]. Thus Artoo was presented in a manner where claims were developed about a specific model, rather than focusing on the process by which claims could be developed

and how different types of evidence can be used to establish those claims. Of critical importance to that process, from which everything else flows, are two simple questions: 1) Has the right model been developed to address the specific question of interest? and 2) has the model been built correctly to address the specific question?

On the surface these might sound like obvious questions to ask and people might be convinced that they have indeed satisfied both questions in a positive manner. However, what is the evidence for such an assertion? If the model developer was asked to provide clear evidence that their model is indeed fit for purpose, what evidence would be presented, and how would that evidence be presented? Consider a number of issues associated with model development: 1- what is the scope of your model in terms of the pharmacological question you intend to ask? 2- who, or what, have you relied on for the underlying evidence to build the model? 3 what assumptions did you make with respect to the biological system you are working on and how it works? 4 what assumptions did you make when moving from understanding your biological system, into mathematics? 5- why did you choose a particular modelling style over another; and there are potentially many more questions that could be asked. Indeed, alongside prompting these questions, adopting such an approach can support inter-team working, having to explain, and document, the rationale behind model development can promote greater transparency in the model itself, and open it to wider scrutiny, which in the longer term, will promote better model development.

These questions are routinely addressed in the area of safety engineering, where ensuring that the correct device has been built, and that the device has been built correctly are potentially of critical importance. Consider a simple example, the airplane. One assumes there are some basic things to get right when building an airplane, for example the need for wings and an engine, but what you build also depends on what the plane is to be used for. Is it a transport plane or a passenger plane? Is it to be used for short distance, or long distance? Ensuring you get the requirements clear ahead of time is important, so understanding the purpose for which the plane is to be used is an essential part of that process. Equally important is ensuring that what was required, was built correctly. Were the right materials used?, was a rigorous engineering process undertaken?, was the plane tested appropriately?, are there instructions on how to use it?, and have you taken appropriate steps to identify and address possible sources of risk? Safety is now taken

for granted by passengers and we are, rightly, assured that safety is a primary concern when building and using aircraft. Often that industry, and others, make use of safety cases through the process of GSN to establish an argument for the safety of a system [13, 15, 18].

Whilst developing and using a QSP model is not the same as building an aircraft, there are analogies between the processes that leads to the construction and application of both. A QSP model might be used as a key decision-making tool in determining dosing regimes or within clinical trials [9, 10], which has potential safety critical implications, or identify avenues of further (expensive) research that might otherwise be avoided. Whilst we might not want to establish a safety case for a model, establishing that a model is fit for the intended purpose for which it has been designed has the potential to increase confidence, transparency and ultimate usage of such models in pharmacological studies [20, 34]. GSN in the context of safety, and now in the context of model development has been developed at York, and as yet is not widely used. However, it is through this tutorial that it is hoped the wider use of such an approach will be adopted.

In this paper we provide a methodology that can be used to robustly develop argumentation structures that examine the rationale employed at various stages of the development of a model. By encompassing all aspects of development, from composition through implementation, analysis, and documentation; this approach provides a methodological structure with potential to increase confidence in the application of computational models as predictive pharmacological tools. Although we ensure the focus is on the argumentation approach, we detail its application in the context of a mathematical model of granuloma formation in the liver [3], a inflammatory immune response that occurs in response to infection with the parasite Leishmania donovani. We show how exploring the rationale behind the development of this simulation and assessing the composition of the model after implementation, eases the assessment of simulation-derived predictions in the context of the purpose for which this model has been designed: to explore potential interventions that could further our understanding of treating this disease.

LEISHMANIASIS AND COMPUTATIONAL MODELS

Visceral leishmaniasis is a systemic tropical disease which, in the absence of treatment, is usually fatal, with 20,000 - 40,000 deaths annually [8]. A defining feature of the immune response to infection with Leishmania donovani parasites is the focal accumulation of inflammatory cells within the liver: these aggregations are known as granulomas and provide a focus for immune mediated elimination of the parasite. The stages of the immune response that follow infection and lead to granuloma formation and eventual parasite clearance are illustrated in Figure S1. Importantly, the cellular composition of the granuloma is dynamic and may comprise monocytes, T cells, and a range of other leukocytes including B, NK, NKT, and dendritic cells in differing numbers and relative proportions [29]. Achieving an appropriate balance between cells that produce pro-inflammatory Th1-type cytokines (e.g.IFN) and regulatory cytokines (e.g. IL-10) is important for stimulating macrophages sufficiently to kill intracellular Leishmania, but without causing an over-exuberant immune response that leads to destructive tissue pathology [29, 33]. Defining how this balance across multiple cell types evolves over time during natural infection and and how it might alter as a consequence of the administration of drugs and other therapies provides a significant challenge in experimental immunology.

To generate insight into this important open question and move towards the development of novel therapeutics against Leishmania donovani, experimental techniques are required that are both less invasive and more ethically achievable than those used to study HVL or EVL. Computational and mathematical approaches permit the development of models that do not share the same constraints, and add capacity to interpret underlying biological data [22] and to provide an experimental tool for exploring new hypotheses that could be examined using traditional experimental approaches [11]. This methodology has previously been employed in the development of a Petri net model of granulomatous inflammation in the liver of mice [3], motivated by the need to develop a tool capable of generating insight into the importance of macrophage deactivation in immune regulation. For the full design, implementation, and analysis detail that underlies this model we refer the reader to the models accompanying publication and supporting materials [3]. To provide a brief overview for the purposes of this tutorial, the Petri net [25] (notation in

Figure S2A)) captures biological entities involved in disease progression and resolution (T cells, phagocytes, NKT cells, NK cells, and the Leishmania parasites) as places that hold a number of counters. These counters signify the levels of each component at a particular time-point of the simulation. Between each place are transitions that move tokens from one place to another, decreasing or increasing the number of tokens as required (specified by different line and arrow combinations, as shown in Figure S2(A). Each transition is designed to capture a biological process, and is a mathematical construct controlled by a number of parameters. At each timepoint the transitions between places fire at a rate determined by probability density functions and the number of tokens in each place. The simulation is designed to capture disease progression and resolution over an extended time period. A high level overview of the leishmania Petri net model is reproduced from [3], in Figure S2(B).

By running the Petri net model under different simulated physiological conditions (parameter exploration), the authors were able to suggest pathways through which regulation of effector functions occur within the granuloma. Yet, for the potential of these insights to further our understanding of the disease and impact therapeutic development to be realised, it is vital that the composition, implementation, and analysis processes through which the model has been developed are transparent and understood.

ENGINEERING TRANSPARENCY

In this section we outline a process using structured argumentation that assists the recording of justifications and rationale for both the biological detail and engineering processes that underlie the development of a computational model. The process and associated tools to support that process take inspiration from the field of safety-critical systems, where it must be demonstrated that a software system is as safe as reasonably practicable [17]. Acceptable safety can be established and presented using arguments over evidence. For increased accessibility and ease of communication, Goal Structuring Notation (GSN) [30, 2] was developed as a visual notation for the presentation of arguments detailing safety cases in critical systems engineering. The role of GSN in the wider safety community is significant with various large industries making contributions to the GSN standard [1].

In exemplifying an approach to expose the rationale underlying the development of a model, we utilise, and suggest the use of, a previously published argumentation tool by ourselves, Artoo [4], that permits the creation of a diagrammatic summary of the structured argument of fitness for purpose. The semantics of the argumentation structure employed in Artoo are inspired by that of GSN, with some modifications introduced to allow an alteration of focus from safety cases to providing a rationale for fitness for purpose. The argument is presented as a tree of connected argument components, of specific shapes (Figure 1). The semantics are detailed in Figure S3. These components start from a top-level claim (a GSN goal). At the beginning of the process a set of fitness-for-purpose requirements (referred to as goals or claims, that the argument seeks to substantiate) should be established, with an accompanying set of strategies that can be used to assess whether the requirement has been met. The strategies typically break down goals into sub-goals, and eventually link to evidence supporting the claim, alongside the source of the evidence where appropriate. If a requirement cannot be fully supported by available evidence, for example where there are gaps in the biological understanding, then the assumptions and abstractions made in place of this evidence are documented, opening all implementation decisions to scrutiny by other researchers in the field and identifying areas of biological study that have been overlooked or require further laboratory work. The process of constructing a claim using the semantics in Artoo is described in Figure S5.

Figure 1: **Caption**

Arguing Fitness for Purpose

As outlined above, whereas GSN is applied to demonstrate evidence in safety cases, our purpose is to develop a fitness for purpose argument with respect to a model. This change in motivation introduces a subtle but important change to the semantics. When arguing over safety, it is critical that a claim is terminated by a suitable evidence node supporting that claim. However, when documenting our rationale that a model is fit for purpose, the construction of an argument may not have a clear ending, in respect of there being no available evidence to substantiate a claim [4]. Where this happens, this should not automatically be seen as a weakness in the model, yet could instead reveal a number of

things. First, that a claim that is believed to be reasonable may in fact not be reasonable at all, and the process of constructing the argument has led to this conclusion. At this point, it might be wise to review the argument, alongside the model to investigate why this might be the case. Second, it might be that the claim is reasonable, but there is no evidence that is acceptable (as defined by the creator of the argument structure). In the case of arguing fitness for purpose, the claim can be left as undeveloped, that is the claim can remain in the argument structure, but highlights a clear gap in the evidence base, thus providing informative transparency of the lack of evidence to support the claim. Such a modification is vital in QSP modelling applications, where expert opinion and assumptions have to be used to mitigate the fact that the understanding of the biological system may be incomplete.

Taking the description in Figure S4 as a template of how to develop a claim, we turn attention to developing claims that encompass all stages encountered in model development. In Figure S5 we have split the process into seven distinct phases, all of which, we believe, greatly benefit from the adoption of a structured argumentation approach in revealing the rationale employed at that stage. To exemplify creation of argumentation at each phase, we now go through each in turn, providing case study examples in the context of leishmaniasis.

Step 1 - Define Purpose of the Model

As can be seen in Figure S5, understanding and defining the intended purpose of a model is a key part of the process, as the rationale for the other key phases of model development is strongly linked to that purpose. Purpose in this context can be defined as for what question the model is intended to answer. This purpose may vary from being a general model intended to explore a range of hypotheses and capture many components, or a very specific model that is intended for a distinct scientific question. In either case, a clear purpose should be defined and a clear scope of the model established, with key questions derived that the model will be used to address. The definition of the purpose forms the first stage in the construction of the argument structure: the top level claim. As described in Figure S3, this top level claim is usually associated with context nodes that define the key terms used to specify that purpose. From here, strategies are then set that will be used to argue that the top level claim is met: that the tool is fit for its specified purpose.

Figure 2 shows the top level of the argumentation structure used to explore the rationale underlying the development of the leishmaniasis model. The purpose of the model is clearly stated: to explore the effects of the cytokine IL-10 on EVL, parasite infection and regulation of granuloma formation. The top level claim is therefore made that the model effectively captures EVL in the liver, thus a useful tool for meeting the intended purpose. Attached to this claim are six strategies that will be used to support the claim. It is hopefully easily noticed that these six claims correspond to the six rounded rectangles in Figure 3: an examination of the rationale of each phase in the process of model development. This section continues with examining each of these sections in turn.

Figure 2: **Caption**

Step 2 - Assess available biological evidence

Once a purpose has been defined, an understanding of the underlying pharmacological and biological processes that will be used for the development of the model needs to be established. It is often at this stage where the scope of the model can be compromised, with the desire to include as much biological information as possible, but possibly at the expense of simplicity (or necessity). Clear rationale for what biological and pharmacological evidence is being used should be produced: without a specification of the data used or any assumptions employed, it is difficult for researchers using model-derived predictions to relate this prediction to their own experimental study. Step 2 of our process supported by argumentation is used to assess (i) the scope of any supplied biological data; (ii) the understanding gleaned from experts studying the biological system and (iii) the areas of understanding that are currently lacking. For each of these, an argumentation claim will be established and an appropriate strategy developed to support the claim. This all contributes to creating the scope of the model. For example, evidence could exist as a log of the experiment that collected the data, or a list of time-points at which the data was collected. Employing this technique ensures that the model developer is aware of the extent to which the current biological system is understood, and the scope of which any data can be included in the developed model.

Figure 3 expands on the known Biology. At this stage of the process we are documenting what has been considered and collecting evidence for mechanisms and species without

making a judgement of whether they will be included in the model - this judgement is made in step 3. The strategy considers the cell populations, cytokines and chemokines that are mentioned in relevant literature. This is useful for generating a list of species that the modeller may later include, or exclude, depending on the weight of evidence for their involvement. Also on the top level is the micro-environment, which if correctly scoped, may exclude populations or mechanisms that fall outside the intended purpose of the model. As an exemplar for the purposes of this tutorial, we have expanded on the cytokines, showing a list of all the cytokines that are considered in the literature. Although the complete argument expands the rationale for inclusion of all cytokines, our exemplar expands on IL-10 and IL-1. For IL-10 it is thought that increasing levels of IL-10 are associated with parasite growth and suppresses parasite clearance [37, 19, 28]. IL-1 is a known pyrogen (meaning that it can cause the host body temperature to rise), and can potentially contribute to parasite killing through heatshock [31].

Figure 3: **Caption**

Step 3 - Rationale for Biological Assumptions

In step 2, consideration is given to the scope of the underlying biology and pharmacokinetics, without consideration of how this will be implemented in any model. However, that step may also have revealed areas of biological understanding that are incomplete, yet need to be included in the model. This can be seen in Figure 4, where the impact of the pyrogen IL-1 is noted as not being fully understood. Where such evidence gaps are identified, well informed, justified, assumptions will need to be introduced into the model. It is critical that the justification for any such assumptions are documented alongside the predictions generated by the model, as their introduction may have an influence on the validity of that prediction. If, for example, the purpose of the model is to produce predictions that inform laboratory research, it is vital that confidence in the assumptions are a fair reflection of the experimental system on which they will be testing this prediction: key when financial and technical resources have to be considered within a study.

In Figure 4 we expand on two examples from the cytokines that were being considered in Step 2. We demonstrate two common simplifying assumptions. For IL-1, the proposed mechanism of action on parasite load is killing of parasites indirectly via heat shock. It

can be argued that heat shock is neither necessary nor sufficient for parasite clearance, as evidenced by the lack of impact of IL-1 receptor blockade on acquired resistance or granuloma formation[33]. Considering the purpose of the model, it is reasonable to assume that IL-1 can be excluded despite the fact that there is some evidence that it could impact parasite load. This exclusion of IL-1 is one type of simplifying assumption. Figure 6 also shows a partially developed argument for merging IFN and TNF which ends in the undeveloped claim that they perform the same function and can be merged into a single proxy species. Both of these simplifying assumptions depend on the stated purpose of the model for their potential validity. Both simplifying assumptions are to some extent judgement calls that multiple stakeholders may wish to examine and influence, which elucidates the importance of transparency and documentation of the argumentation.

Figure 4: **Caption**

Step 4 - Rationale for Modelling Approach

In implementing any model of a biological system, there may be several techniques that could be selected (i.e. modelling paradigms, software tools). In this step, the model developer can use argumentation to justify the engineering decisions taken during model implementation. There can be a temptation to choose the modelling tool of convenience, one that a modeller is familiar with, however, this can be a mistake. It is well known that different modelling techniques can show different types of results and have an effect on what is observed [14]. Therefore, it is important that the rationale for the choice of modelling system be exposed. As an example, a claim could be made that an agent-based modelling paradigm is most suitable for addressing the question of concern. Strategies would then be employed to determine whether this is indeed the case, or whether other approaches such as Ordinary Differential Equation (ODE) modelling would be more appropriate. By using argumentation at this stage, the developer has a record of the implementation decisions that were taken, with a fully evidenced justification of why these decisions were taken.

Figure 5 shows a subsection of the argument concerning the modelling approach adopted in the development of the Leishmaniasis simulation. From the top claim specified in Figure 2, the strategy is to argue the appropriateness of the adopted approach, in this

case stochastic Petri nets. From here, our claim is that the adopted paradigm provides the means to represent the required aspects of the biological system. To support this claim, one would be required to compare the available approaches, and as such the stated strategies involve examining implementing the model as a Petri net, agent-based model, or ODEs. For the scope of this tutorial, Figure 5 expands on the Petri net suitability claim, arguing that we can capture the required stochasticity, capture granuloma heterogeneity, handle small integer number calculation, and produce an implementation that is computationally tractable. In this case we are able to evidence all four claims, suggesting we have a suitable approach for capturing the key aspects specified in the claim.

Figure 5: **Caption**

Step 5 - Rationale for Modelling Assumptions

By employing steps 2 and 3, any gaps in the biological understanding became apparent and were addressed via appropriately justified and documented assumptions. Previously we described how critical these assumptions were when relating the simulated system to the real system of interest. Additionally, this critical issue is also applicable when introducing simplifications that may be made during the development of the model. At this stage, it may be sensible to determine whether the full extent of the biological system of interest scoped in step 2 needs to be captured in the model. For example, modelling the impact of a number of cell receptors and their respective chemokines could potentially be reduced to a model of a single proxy chemokine and receptor pair, if what is being examined is the higher-level effect produced by these chemokines and receptors as an ensemble. An example of a similar issue could be a biological system consisting of tens of thousands of cells: complexity that may not be tractable to simulate. The simulation developer may determine that only capturing a percentage of that environment is enough to understand the overall emergent behaviour of that system. Taking a number of biological concepts and simplifying these into a single mechanism, or determining a biological concept to be unnecessary given the scope of the model, does however introduce assumptions that must both be taken into consideration when relating a model-derived result to the real system and be justified.

Figure 6 shows a subset of the argumentation structure produced from the top level

strategy to argue over the modelling abstractions. Similarly to previous examinations of the biological information and assumptions, here, claims are made concerning the appropriate capture of the cells, cytokines, chemokines, and the environment. For the scope of this tutorial, we have included the argument of one key assumption in the model: that the dynamics of monocyte derived macrophages, dendritic cells, and neutrophils can be adequately captured by a single cell type. Such an assumption reduces the complexity of the model, yet could impact the meaning of any results generated. As such we support this simplification with two claims: that parasites are not observed to replicate in these cell types, yet these cells contribute to the cytokine microenvironment in the granuloma. The first, supported by collaborators opinion, would suggest that these cells could potentially be abstracted out of the model altogether, as they do not influence the models purpose. However this is contradicted by the second, which makes the claim that these cells contribute to the cytokine environment of interest. As such, we argue that these are required, but can be abstracted to a single proxy cell type that expresses the cytokines identified in Figure 3.

Figure 6: **Caption**

Step 6 - Engineering the Implementation

When going through this process alongside the development of a simulation, the developer will now have justified the modelling approaches they are going to use (step 4) and the abstractions they will make in implementation (step 5). The next step is to implement the model. Issues of trust in simulations for science have previously been raised, and much has been written on how this could be countered by the release of code [27, 26, 24]. However, we believe our approach to structured argumentation also provides a means of increasing trust in the implementation alongside such arguments. Argumentation could, for example, be used to argue that the code meets the specifications developed in the previous phases above, and that an adequate testing routine has been developed and performed.

Figure 7 shows a subset of such an argumentation structure for the Leishmaniasis simulation, arguing that the system meets requirements for implementation and has been adequately tested. The former is in some respects easier to show: claims can be made

concerning particular biological behaviours that are evidenced by aspects of the model (such as equations), and links can be drawn to evidence derived on argumentation diagrams from previous phases of the process. Testing a complex simulation is much more difficult. In Figure 7, the strategy to argue that the Leishmaniasis simulation was adequately tested has been to ensure adequate structural coverage of the code by tests. In this case, as is typically the case in high integrity software engineering, this strategy is split into three phases: requirements testing [36]; unit testing [23]; and manual review.

Requirements testing ensures that the system has a collection of requirements describing the tasks that the system should perform, and it ensures that each requirement has an associated test (or collection of tests) that demonstrates the system fulfilling the requirement. The requirements tests are run through the implementation to check that they pass and to measure their structural code coverage. If all the requirements tests pass, then this demonstrates that the implementation performs its tasks correctly. If all the requirements have appropriate tests that pass, then this demonstrates that the implementation performs the correct tasks. If the requirements tests produce full code coverage, then this demonstrates that the implementation performs only its tasks and nothing else.

In practice, it might be impractical to achieve full code coverage using just requirements tests at the system level. For example: there might be some error-checking code deep within the call tree that is difficult to trigger under normal conditions. For these cases, unit tests are used to inject particular values into the implementation to increase the code coverage of the requirements tests.

Even using unit tests, it may not be possible to achieve full code coverage for some types of code. For example: robustness checks, system libraries, or code that only executes when running the system in a different mode. For these cases, the code is reviewed manually to either determine that it will not execute in the situations we are providing, or to argue why it does not need to be tested (for example, a commonly used system library). Given the criticality of models we consider adequate testing to mean achieving 90% statement coverage and 90% branch coverage through requirements tests and unit tests, with the remaining code reviewed manually.

Figure 7: **Caption**

Step 7 - Justify experimental approach / analysis

Once a simulation has been designed and implemented, model developers will perform in silico experimentation and statistical analyses designed to elucidate biological insight from the model [6]. However, for full transparency, the model developer should adopt an argumentation approach to argue that the experiment is necessary and designed correctly, prior to any simulation runs being performed. This will ensure that the time spent on running complex simulations is minimised, and also ensure the analysis routines take into account implementation inherent issues such as the inclusion of stochastic behaviours. Results from the experiments and the analysis techniques employed to fully understand the behaviour of a model need to be interpreted in terms of (i) the scope of the designed simulation; (ii) the biological system being studied. The final stage of our process uses evidence-based argumentation to draw conclusions from simulation-derived results, utilising the evidence compiled in Steps 1-5. Here, the simulation developer may make a claim regarding some insight generated during the modelling project. They may then draw on evidence from the complete argumentation process to show that the generated insight can be supported. Figure 10 shows a subset of the argument that the experimental analyses performed are well designed and appropriate. This is divided into sub-claims that describe two sets of experiments: (i) statistical analyses employed to understand the behaviour of the model, and (ii) in silico experimentation used to perform experiments that may be difficult to perform in the laboratory. Both sets of experiments are detailed in [3]. Figure 8 shows one of each: appropriate sensitivity analyses for the first and IL-10 related experimentation for the second. In both cases the claims are supported by the reasoning for the particular experiment, the experimental strategy, and the results. By ensuring the design of such analyses is transparent, others using the result in their own context are clear as to how each prediction has been derived.

Figure 8: **Caption**

USING ARGUMENTATION TO REFINE MODEL PURPOSE, DESIGN and IMPLEMENTATION

Where the described process is employed, the key biological information to be modelled will be identified, translated into a format that can be encapsulated within a computer code, and developed into a computational model through which predictive experimentation can be performed. Completing a process where each step on this path is justified and documented is advantageous in determining the degree to which predictions made can be related to the real-world system being studied [4]. Whereas the process described above focuses on that process of exploring the rationale of a model either during construction or retrospectively, a completed argumentation structure should however not be seen as a static document, and offers further advantages in cases where a model is to be repurposed or refined.

As an example, consider the Leishmaniasis simulation that has been used as a case study throughout this tutorial. This model captures the processes within EVL, an experimental mouse model of visceral leishmaniasis. However, the overriding objective is to further our understanding of Leishmania in order to expedite the development of novel therapeutics against the disease in humans. Although it is generally accepted that the mouse provides an adequate model for exploring the disease in humans, this remains a model of the disease in the mouse, and the links between this model and the human disease need to be understood. One potential strategy could be to repurpose the model: altering the focus to capture HVL rather than EVL. If this were to be undertaken, possessing a rationale for the design, construction, and analysis of the computational model of EVL would be very useful in determining the extent to which the model needs to be altered to capture HVL. For example, an assessment of the biological information on which the EVL model was constructed (step 2) and the assumptions that were introduced in that model (step 3) would determine the relevance of that data to any model of HVL. Where argumentation was used to construct the original model, we also argue that the approach could be very useful in arguing over any alterations that are made if the purpose of the model is adjusted.

Additionally, possessing a complete rationale detailing model development and analysis could be advantageous in assessing the composition of the resultant model. Following

a detailed exploration of the biological information, addition of necessary engineering assumptions and abstractions, and implementation of the computational tool, the Leishmania Petri net model comprised 174 transitions between places, with each transition designed to capture a particular biological pathway. Although the authors were able to show that the model could recapitulate the progression of the disease, in comparison to a laboratory experimental model, and predict cellular composition within granulomas [3], no analysis has been previously undertaken as to the necessity of each of the 174 transitions in the model. Such an analysis has the potential to infer further information regarding the key biological pathways involved in disease progression and immune system regulation. From an engineering perspective, the most computationally intensive process in running a Petri net model is initialising each of the transitions: if a number of these transitions were found to be unnecessary, there is thus potential for a large increase in simulation performance.

To examine the impact of each of the 174 transitions, we modified the Petri net model such that the simulation recorded the number of times each transition fired. As the firing of a transition is potentially dependent on the initial conditions and parameter values, we ran the model under a number of initial conditions, over the parameter ranges originally explored by [3]. To ensure adequate coverage of the parameter space, we utilised the ASPASIA sensitivity analysis toolkit [16] to generate 600 sets of parameter value combinations using latin-hypercube sampling [32, 35]. By executing the Petri-Net model under each of the 600 conditions, we were able to determine the number of times each transition fired across the parameter space. Where a transition was found not to fire for any set of initial conditions, one could question the necessity of including this pathway in the model.

This analysis identified 47 of the 174 transitions between Petri net places that were never fired (28%), suggesting a number of the transitions could potentially be removed. Although this would reduce the computational complexity of the model, making simulated analyses and experiments faster, it is important that we understand the impact this change has in terms of our understanding of what the model captures. The argumentation constructed in the development or analysis of a model provides a tool through which any impact can be assessed. Of these 47 transitions that are related to T-Cells, NKT, and NK cells, the majority of non-firing transitions are found to control the silencing of cells due

to a lack of a certain cytokine and reprise of cytokine production due to an increase in environmental cytokine levels. This would suggest that the simulation is never reaching thresholds where these cells are transitioning states. Knowing this, it becomes possible to read through the argumentation to determine if this cell behaviour could emerge from the manner in which the model has been constructed, or whether this is an error. This result could also assist conversations with collaborating biologists, and provide insight into the composition of the granuloma environment.

DISCUSSION

Technological advancements and a focus on interdisciplinarity has resulted in an increased prevalence of laboratory studies being paired with computational modelling research, motivated by the potential to reduce animal experimentation, reduce costs, and perform experimentation that is not possible in the laboratory or informs future clinical studies. However, for computational modelling studies to achieve that potential, it is critical that the relationship between the model and the biological system being captured is fully understood. Any researcher would need to have a high level of confidence in a model-derived prediction before seeking to invest time, expertise, and financial resources into investigating that prediction further in the real system.

The notion of increasing confidence in the application of computational models in biological research is not new however, yet has tended to focus on the end result: the implementation [27]. Such focus has led the field to suggest open-source code [26], that is potentially checked by third-parties [24], and included alongside publications describing that model [12]. However, the issue of confidence in a model must go further than that: the code may well be adequate to do the job it has been designed to do, this does not imply that the biological system has been captured appropriately [5].

In this tutorial we have detailed a process through which the rationale underlying the design, implementation, and analysis of a model of a biological system is generated. We see this process being applied either within a process of model construction or as a tool through which an assessment of a previously developed model can be performed. This process begins by examining the purpose of the study: what it is that the model will be used for. This establishes the scope prior to any experimental work, to ensure

the tool is not being used to generate predictions for which it has not been designed. This purpose is then a key consideration in an examination of each component phase of model development: assessing the biological data; making necessary assumptions in place of a lack of information; choosing the correct modelling paradigm; introducing necessary modelling assumptions; engineering the computational model, and performing experimentation using the tool. Any omissions or ambiguities inherent in any of these phases could impact the potential to relate a model prediction to the real-world: for this to be detected, all design decisions must be transparent.

Adverse outcome pathway (AOP) tools have found application in toxicology and in studies of human risk assessment, providing a means to specify how interactions at the molecular, cellular and organ level can be linked to an adverse outcome [38]. Presented as a flow diagram, AOPs can show the strength of evidence supporting the events in the outcome pathway, yet have come under criticism for splitting the representation of the process from the evidence, providing a simplistic representation of the toxicological process [38]. More generally, yet applicable to QSP-related models, the ODD (Overview, Design concepts, Details) protocol does permit the specification of the purpose behind the creation of a model, the inclusion of biological components, and modules describing the implementation of biological behaviour, alongside relevant assumptions [21]. The focus of ODD is scientific repeatability, rather than fitness for purpose as specified in this tutorial, and lacks the recording of model experimentation and statistical analyses, and motivation for performing those experiments [21]. In producing this tutorial we are not hoping to replace either technique: argumentation could be used alongside either, but we do contend that neither method provides the complete set of information required to convince researchers that a model is appropriately constructed and analysed to meet its intended purpose.

We believe that arguing over the rationale for each of the model development phases identified in Figure 3 can provide a transparent evidence base upon which the contribution of a computational model can be assessed. Alongside a description of the process involved in examining the rationale at each phase, we have shown an example application of the process in examining the rationale underlying the development of a model of Leishmaniasis: developed to further understand this neglected tropical disease to generate insights that could inform future therapeutic studies [3]. In addition to exposing the rationale

behind this model, we then described how this argument could potentially be used to determine the links between this model and human visceral leishmaniasis (HVL), and how the argument could be useful in examining the composition of the model with respect to computational complexity. The approach offers more than a process to be employed in model development or assessment, and is advantageous in redefining the purpose of, or refining the composition of, models developed for QSP studies. Where a computational model is closely tied to a mouse study, structured argumentation using the approach detailed in this tutorial has the potential to provide a robust way of understanding how the model could be repurposed for human studies that predate or inform clinical trials.

Acknowledgements

This work is part funded by the Crack-IT programme, grant number NC/C013117/1 and NC/C013205/1. PMK would also like to acknowledge support from MRC Programme Grant G1000230. JT would like to acknowledge support from The Royal Academy of Engineering and The Royal Society.

References

[1] Gsn working group. Accessed: 2016-09-18.

[2] Combining software evidence arguments and assurance. In *Proceedings of ICSE-2005: Workshop on Realising Evidence Based Software Engineering* (2005).

[3] Albergante, L., Timmis, J., Beattie, L., and Kaye, P. M. A Petri net model of granulomatous inflammation: implications for IL-10 mediated control of Leishmania donovani infection. *PLoS computational biology 9*, 11 (jan 2013), e1003334.

[4] Alden, K., Andrews, P. S., Polack, F. A. C., Veiga-Fernandes, H., Timmis, J., and Coles, M. C. Using Argument Notation to Engineer Biological Simulations with Increased Confidence. *Journal of the Royal Society Interface 12*, 105 (2015).

[5] Alden, K., and Read, M. Computing: Scientific software needs quality control. *Nature 502* (2013), 448.

[6] Alden, K., Read, M., Timmis, J., Andrews, P. S., Veiga-Fernandes, H., and Coles, M. C. Spartan: A Comprehensive Tool for Understanding Uncertainty in Simulations of Biological Systems. *PLoS computational biology 9*, 2 (2013).

[7] Alden, K., Timmis, J., Andrews, P. S., Veiga-Fernandes, H., and Coles, M. C. Pairing experimentation and computational modelling to understand the role of tissue inducer cells in the development of lymphoid organs. *Frontiers in Immunology 3* (2012), 1–20.

[8] Alvar, J., Vlez, I. D., Bern, C., Herrero, M., Desjeux, P., Cano, J., Jannin, J., Boer, M. d., and the WHO Leishmaniasis Control Team. Leishmaniasis worldwide and global estimates of its incidence. *PLoS ONE 7*, 5 (05 2012), 1–12.

[9] An, G. Agent-Based Computer Simulation and SIRS: building a bridge between basic science and clinical trials. *Shock 16*, 4 (2001), 266–273.

[10] An, G. Concepts for developing a collaborative in silico model of the acute inflammatory response using agent-based modeling. *Journal of critical care 21*, 1 (mar 2006), 105–10; discussion 110–1.

[11] Andrews, P. S., Polack, F., Sampson, A. T., Timmis, J., Scott, L., and Coles, M. Simulating biology : towards understanding what the simulation shows. In *Proceedings of the 2008 Workshop on Complex Systems Modelling and Simulation* (2008), pp. 93–123.

[12] Barnes, N. Publish your computer code: it is good enough. *Nature 467* (2010), 753.

[13] Chinneck, P., Pumfrey, D., and McDermid, J. The heat/act preliminary safety case: A case study in the use of goal structuring notation. In *Proceedings of the 9th Australian Workshop on Safety Critical Systems and Software - Volume 47* (Darlinghurst, Australia, Australia, 2004), SCS '04, Australian Computer Society, Inc., pp. 33–41.

[14] Cilfone, N. A., Kirschner, D. E., and Linderman, J. J. Strategies for efficient numerical implementation of hybrid multi-scale agent-based models to describe biological systems. *Cellular and Molecular Bioengineering 8*, 1 (2015), 119–136.

[15] Denney, E., Pai, G., and Habli, I. Towards measurement of confidence in safety cases. In *2011 International Symposium on Empirical Software Engineering and Measurement* (Sept 2011), pp. 380–383.

[16] Dyson, S., Alden, K., Cucurull-Sanchez, L., Larmine, C., Coles, M., Kullberg, M., and Timmis, J. Aspasia: A toolkit for evaluating the effects of biological interventions on sbml model behaviour. *In Review*.

[17] Executive, H., and Safety. *Reducing risks, protecting people.* Crown, 2001.

[18] Fenn, J., Hawkins, R., Williams, P., and Kelly, T. *The Safety of Systems: Proceedings of the Fifteenth Safety-critical Systems Symposium, Bristol, UK, 13–15 February 2007.* Springer London, London, 2007, ch. Safety Case Composition Using Contracts - Refinements based on Feedback from an Industrial Case Study, pp. 133–146.

[19] Gazzinelli, R., Oswald, I., James, S., and Sher, A. Il-10 inhibits parasite killing and nitrogen oxide production by ifn-gamma-activated macrophages. *Journal of Immunology*, 148 (1992), 1792–1796.

[20] Ghetiu, T., Polack, F. A. C., and Bown, J. Argument-driven Validation of Computer Simulations A Necessity Rather Than an Option. In *VALID 2010: The Second International Conference on Advances in System Testing and Validation Lifecycle* (2010), pp. 1–4.

[21] Grimm, V., Berger, U., Bastiansen, F., Eliassen, S., Ginot, V., Giske, J., Goss-Custard, J., Grand, T., Heinz, S. K., Huse, G., Huth, A., Jepsen, J. U., Jørgensen, C., Mooij, W. M., Müller, B., Peer, G., Piou, C., Railsback, S. F., Robbins, A. M., Robbins, M. M., Rossmanith, E., Rüger, N., Strand, E., Souissi, S., Stillman, R. a., Vabø, R., Visser, U., and DeAngelis, D. L. A standard protocol for describing individual-based and agent-based models. *Ecological Modelling 198*, 1-2 (sep 2006), 115–126.

[22] Guo, Z., and Tay, J. C. A Comparative Study on Modeling Strategies for Immune System Dynamics Under HIV-1 Infection. In *Artificial Immune Systems, 4th International Conference, ICARIS 2005, LNCS 3627* (2005), Springer, pp. 220 – 233.

[23] Hamill, P. *Unit Test Frameworks: Tools for High-Quality Software Development.* " O'Reilly Media, Inc.", 2004.

[24] Hayden, E. C. Mozilla plan seeks to debug scientific code. *Nature 501*, 472 (2013).

[25] I, K., W, R., and F, S. *Modeling in Systems Biology: The Petri Net Approach.* Springer, 2011.

[26] Ince, D. C., Hatton, L., and Graham-Cumming, J. The case for open computer programs. *Nature 482*, 7386 (feb 2012), 485–8.

[27] Joppa, L. N., McInerny, G., Harper, R., Salido, L., Takeda, K., O'Hara, K., Gavaghan, D., and Emmott, S. Troubling Trends In Scientific Software Use. *Science 340* (2013), 814–815.

[28] Kane, M., and Mosser, D. The role of il-10 in promoting disease progression in leishmaniasis. *Journal of Immunology 166* (2001), 1141–1147.

[29] Kaye, P. M., Svensson, M., Ato, M., Maroof, A., Polley, R., Stager, S., Zubairi, S., and Engwerda, C. R. The immunopathology of experimental visceral leishmaniasis. *Immunological Reviews 201*, 1 (2004), 239–253.

[30] Kelly, T. P. *Arguing Safety A Systematic Approach to Managing Safety Cases.* PhD thesis, University of York, YCST 99/05, 1999.

[31] McCall, L.-I., and Matlashewski, G. Involvement of the leishmania donovani virulence factor {A2} in protection against heat and oxidative stress. *Experimental Parasitology 132*, 2 (2012), 109 – 115.

[32] McKay, M. D., Beckman, R. J., and Conover, W. J. A comparison of three methods for selecting values of input variables in the analysis of output from a computer code. *Techometrics 21* (1979), 239–245.

[33] Murray, H. W. Tissue granuloma structure-function in experimental visceral leishmaniasis. *International Journal of Experimental Pathology 82*, 5 (2001), 249–267.

[34] Polack, F. A. C., Droop, A., Garnett, P., Ghetiu, T., and Stepney, S. Simulation validation : exploring the suitability of a simulation of cell division and differentiation in the prostate. In *CoSMoS 2011: Proceedings of the 2011 Workshop on Complex Systems Modelling and Simulation* (2011).

[35] Read, M., Andrews, P. S., Timmis, J., and Kumar, V. Techniques for Grounding Agent-Based Simulations in the Real Domain : a case study in Experimental Autoimmune Encephalomyelitis. *Mathematical and Computer Modelling of Dynamical Systems 18*, 1 (2012), 67–86.

[36] Rosenberg, L. H., Theodore, P., Hammer, F., and Huffman, L. L. Requirements, testing and metrics. In *In 15th Annual Pacific Northwest Software Quality Conference* (1998).

[37] Verma, S., Kumar, R., Katara, G. K., Singh, L. C., Negi, N. S., Ramesh, V., and Salotra, P. Quantification of parasite load in clinical samples of leishmaniasis patients: Il-10 level correlates with parasite load in visceral leishmaniasis. *PLoS ONE 5*, 4 (04 2010), 1–8.

[38] Vinken, M. The adverse outcome pathway concept: a pragmatic tool in toxicology. *Toxicology 312* (oct 2013), 158–65.

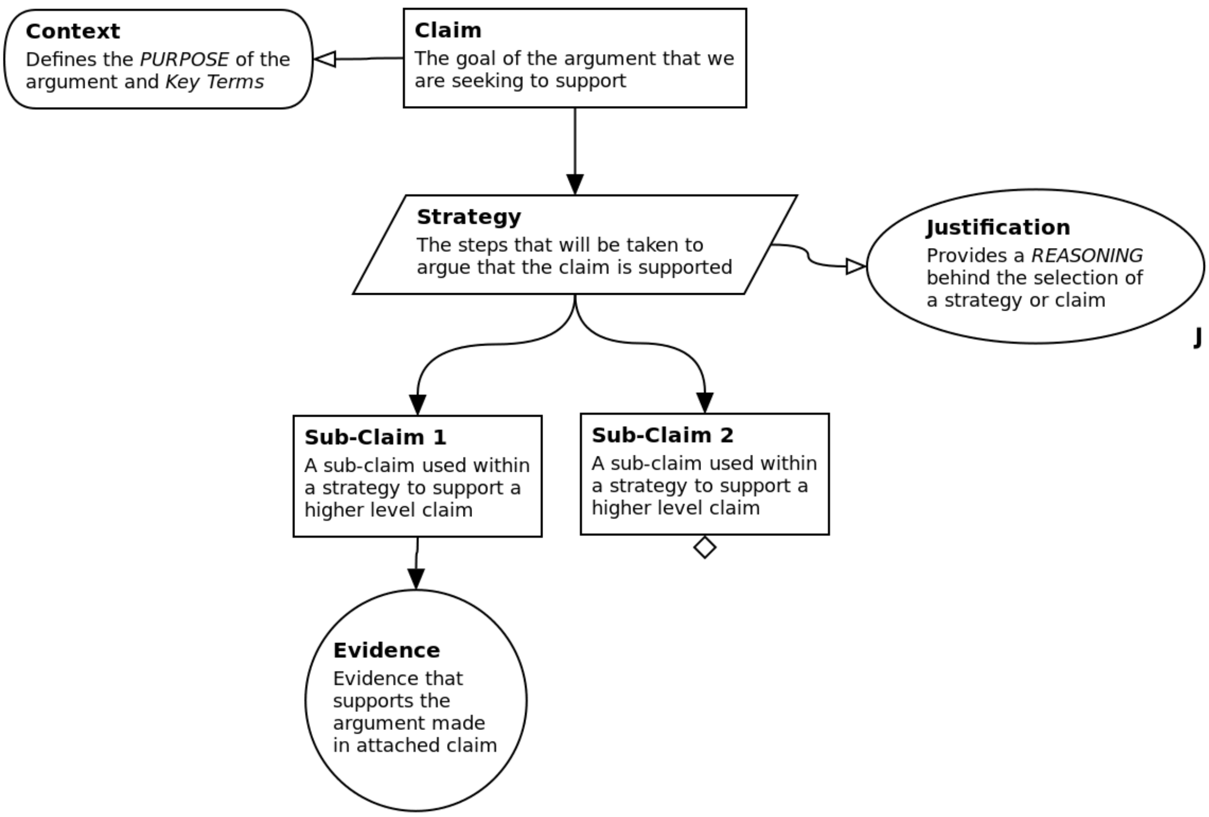

Figure 1: Description of the notation used in the creation of an argument. To show how these components are linked together, we present the description of each component within the format of an argument structure.

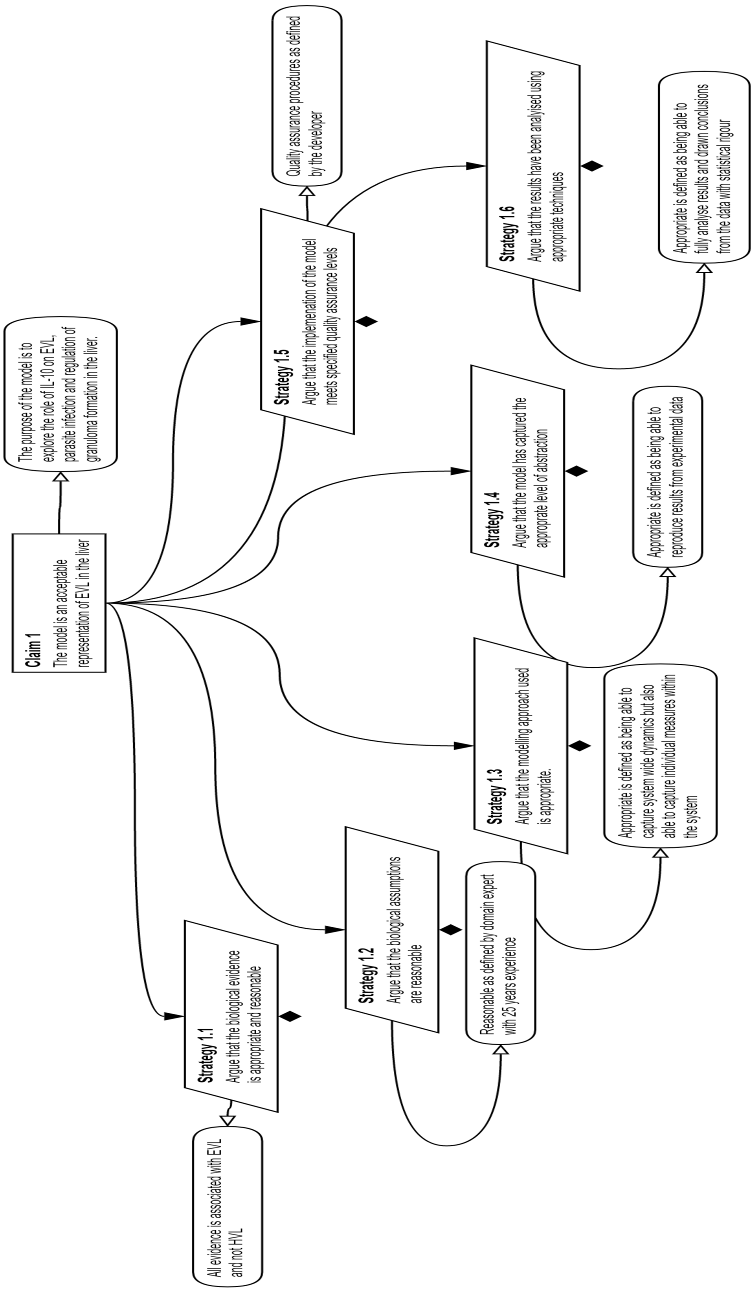

Figure 2: Top level argument in the process of arguing that the leishmaniasis simulation is fit for purpose. Black diamonds indicate the strategies in this figure are expanded upon below.

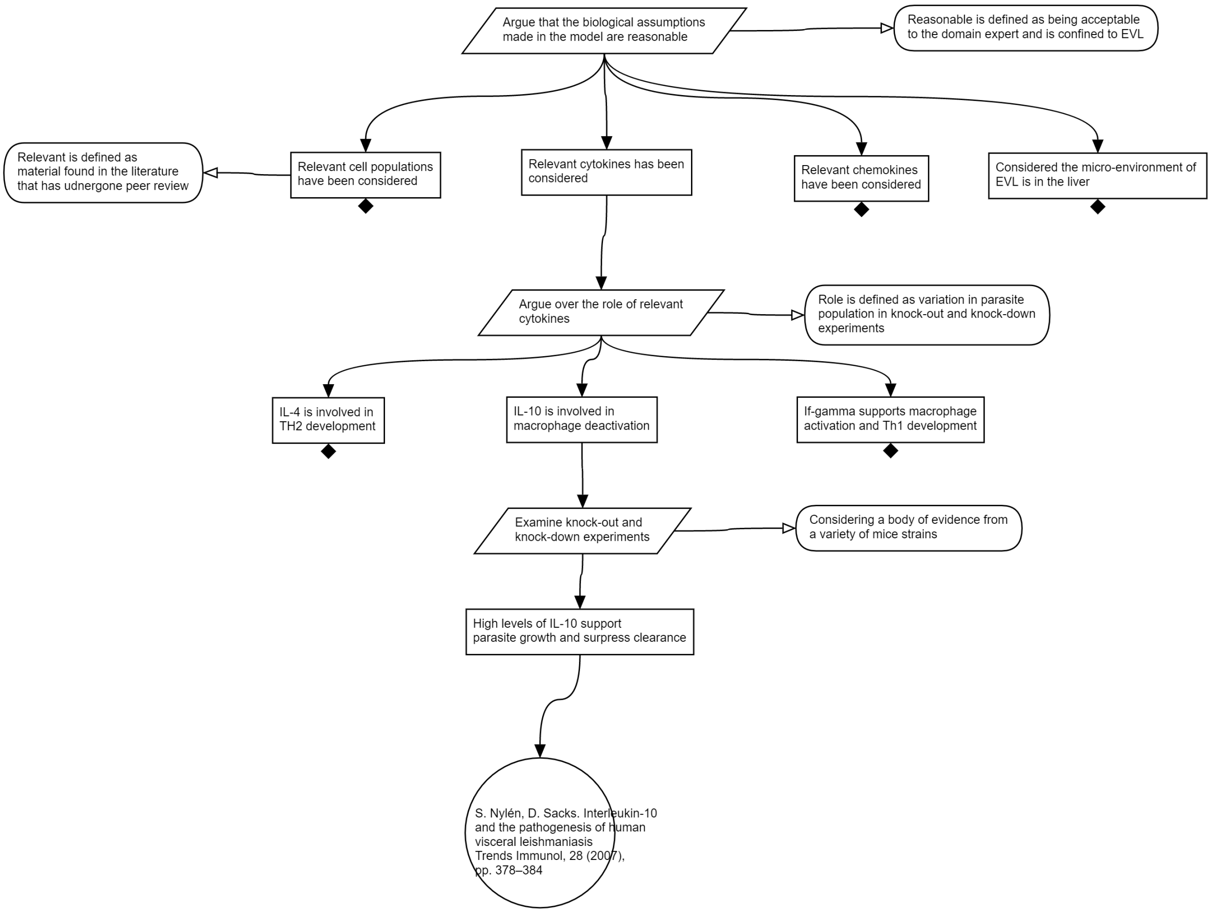

Figure 3: Arguing appropriateness of evidence used as a basis forthe Leishmaniasis Simulation in [3].

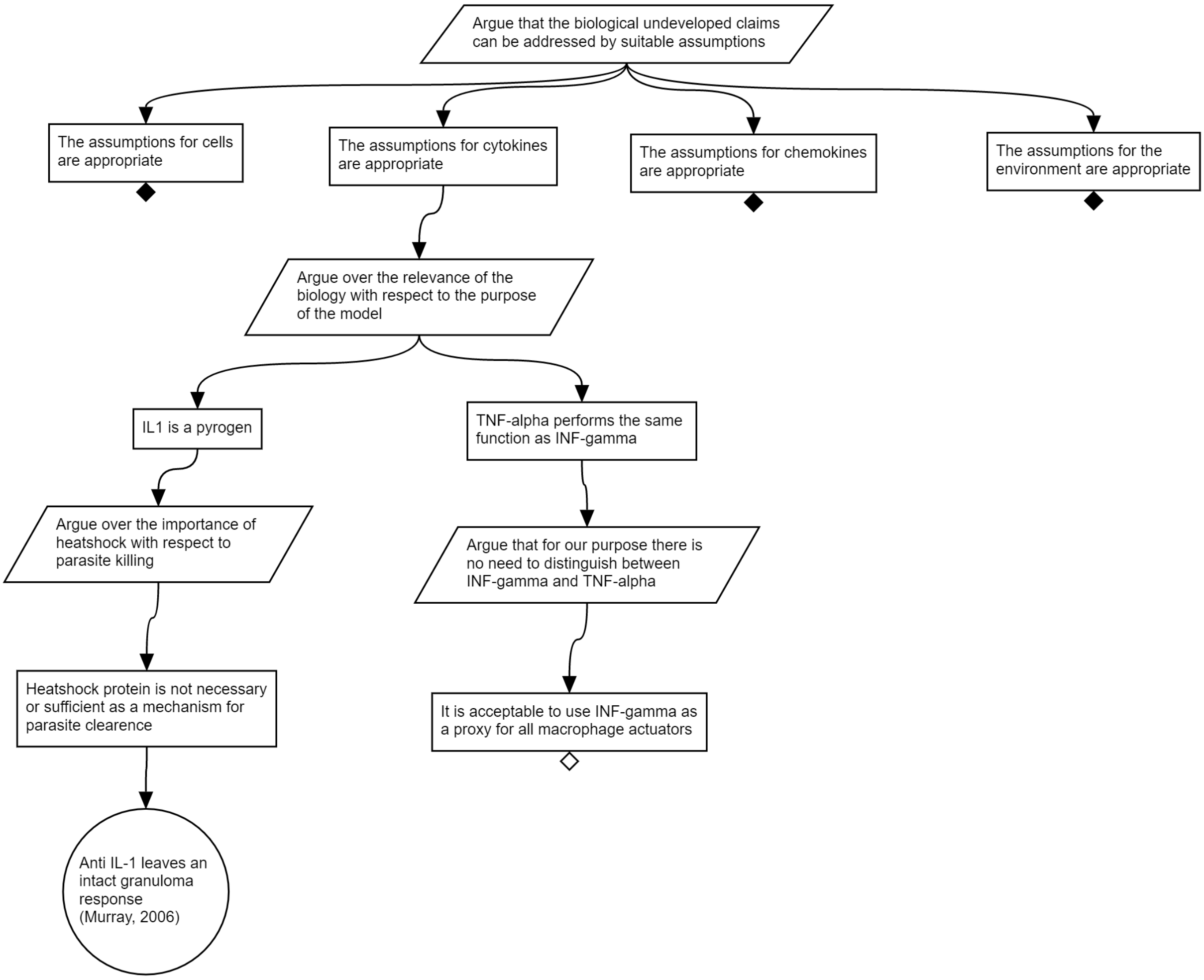

Figure 4: Argument that the biological abstractions introduced in the model are suitable. In this case, the approach is exemplified by focusing on abstractions of cell type to be included in the model.

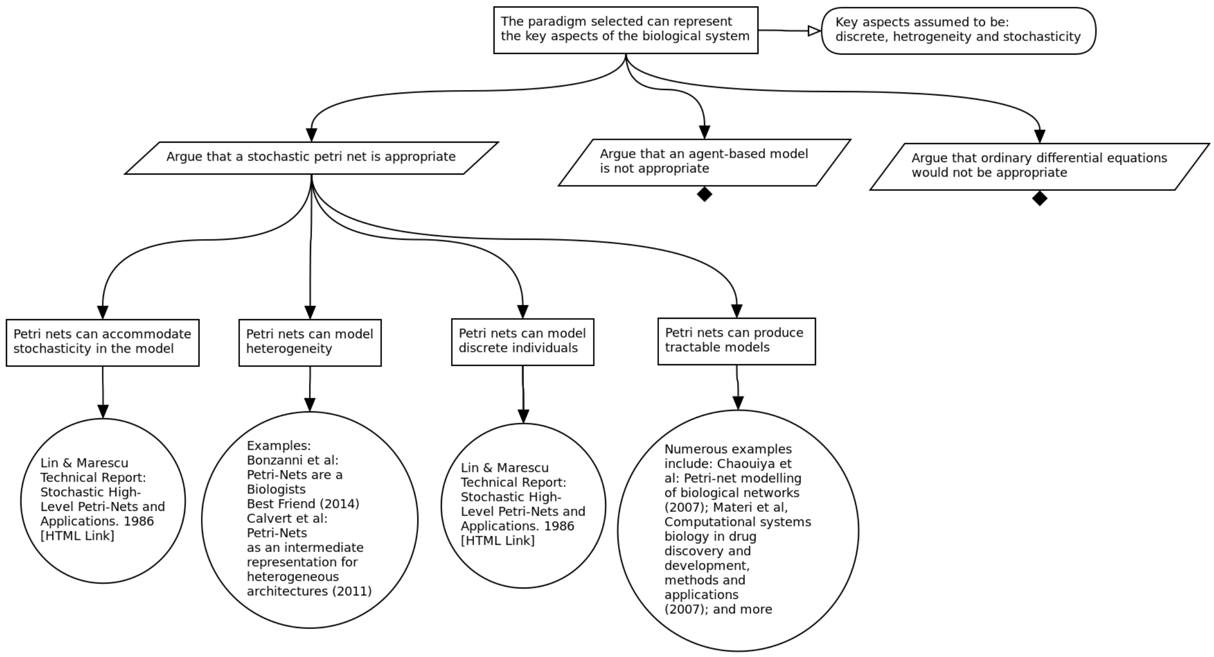

Figure 5: Argument that the adopted modelling approach is adequate given the research context. In this case, the approach is exemplified by focusing on the choice of modelling paradigm: Petri Net, Agent-Based model, or ODEs.

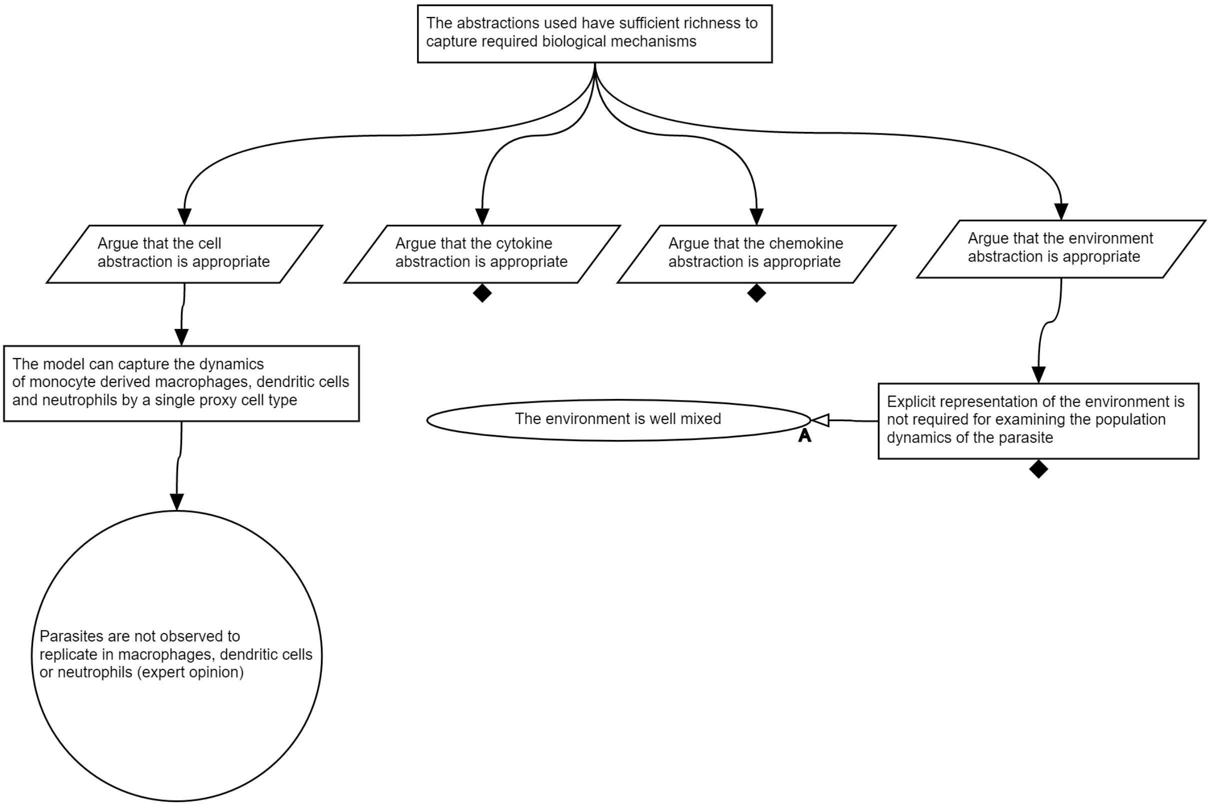

Figure 6: Subset of the argument that supports the rationale for abstracting modelling abstractions.

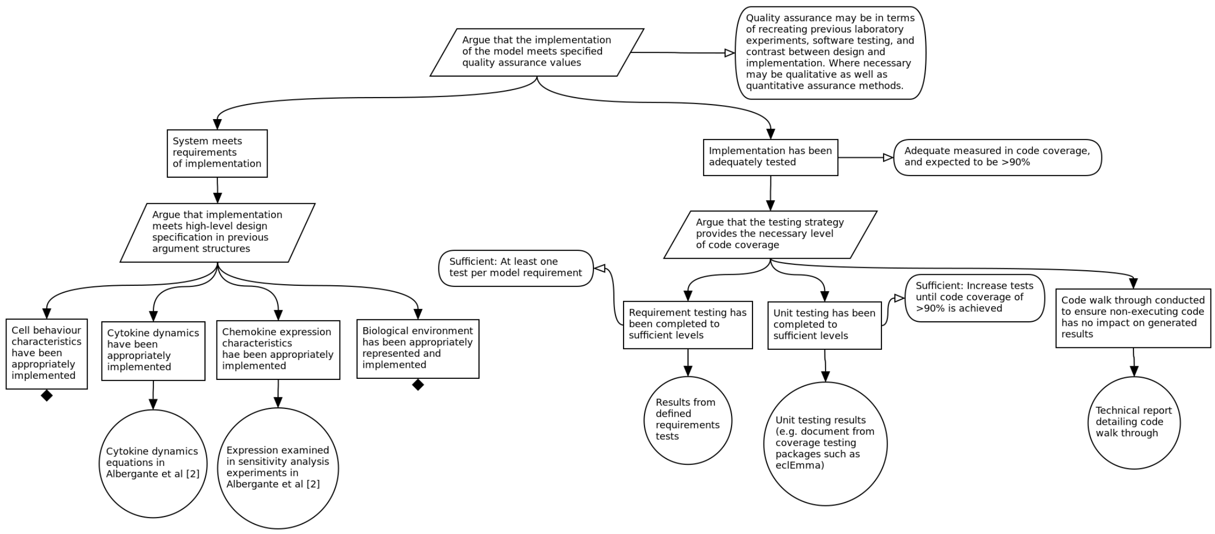

Figure 7: Subset of the argumentation structure used to argue that the implementation of the model is adequate for meeting the purpose specified in Figure 2.

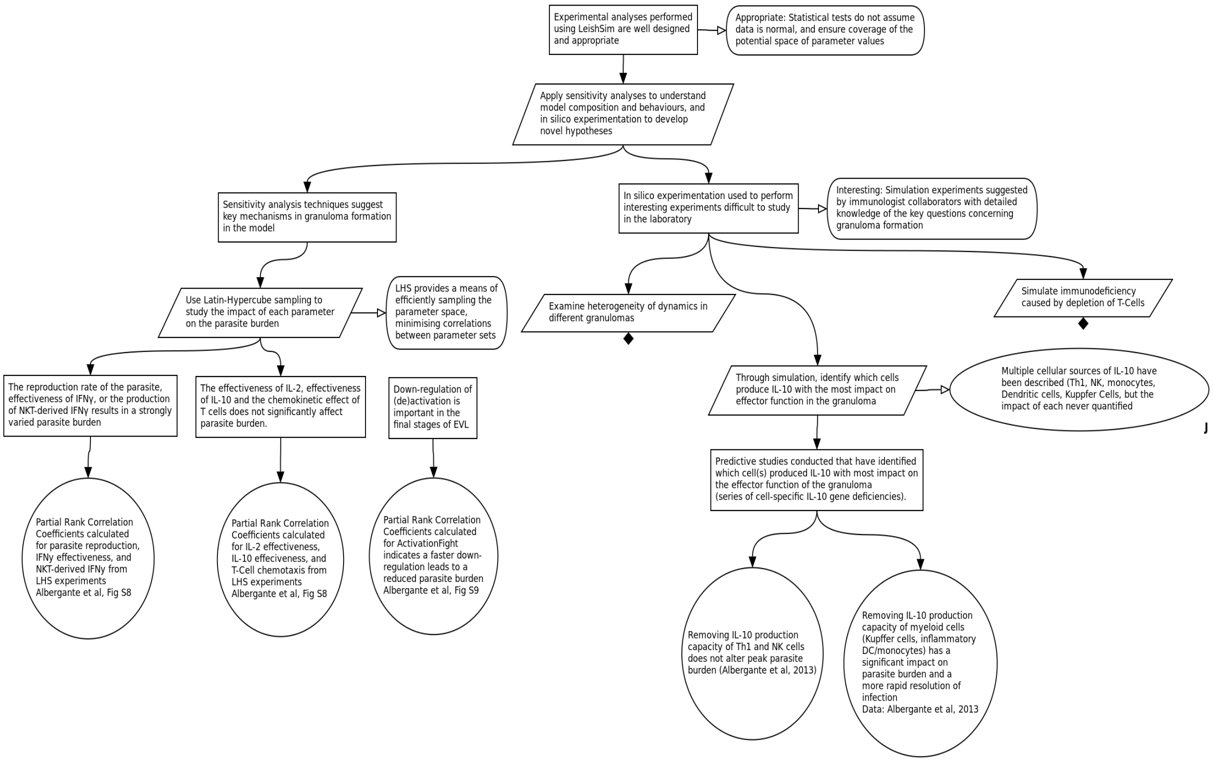

Figure 8: Subset of the argumentation structure for the design of the experimental analysis.

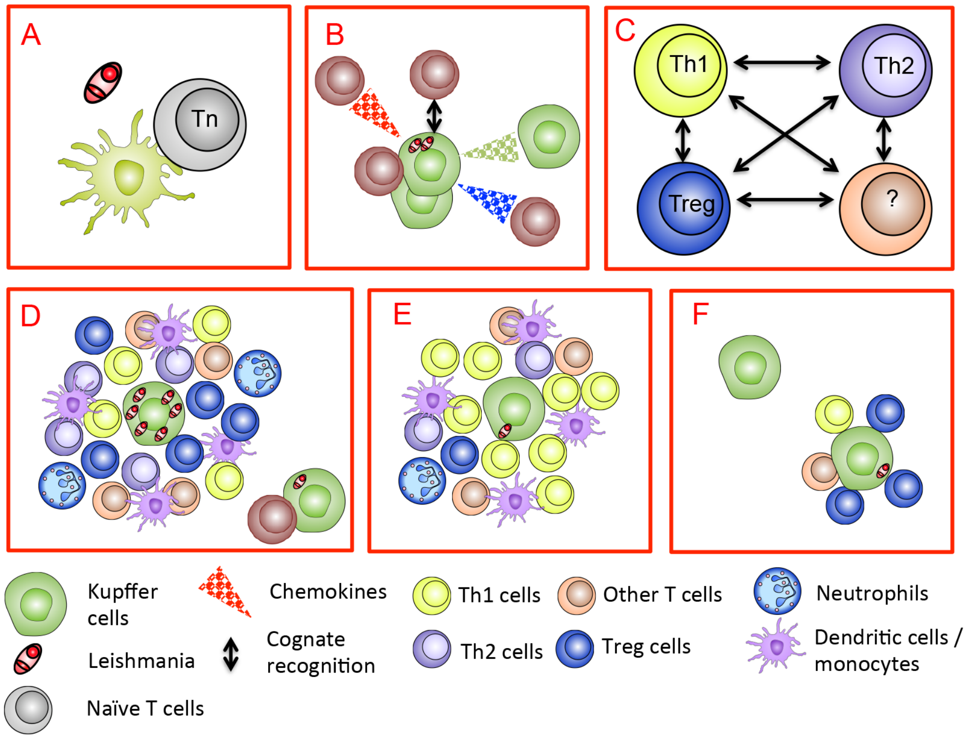

Figure S1: Summary of immune response associated with granuloma formation in leishmaniasis. A. Within hours of experimental infection with Leishmania donovani, dendritic cells present parasite antigens to nave T lymphocytes in lymphoid tissues to initiate an adaptive immune response. B. Simultaneously, parasites in the liver infect resident liver macrophages (Kupffer cells), stimulating the production of chemokines that attracts innate lymphoid cells (of which NKT cells are best characterized). NKT cells engage with infected Kupffer cells via cognate receptor-ligand interactions, amplifying the chemokine response to attract additional Kupffer cells, NKT cells and eventually other cell types (see D, below). C. Over the first few days of infection, T cells differentiate into a variety of subsets (Th1, Th2, Treg), producing cytokines that may cross-inhibit or cross-stimulate T cell differentiation. These cytokines also promote (e.g. IFN) or inhibit (e.g. IL-10) the ability of macrophages to kill Leishmania. D. The relative balance of different T cell subsets, together with monocytes, dendritic cells, and occasionally neutrophils that are attracted to the expanding granuloma determines parasite burden. Notably, granuloma development is asynchronous (lower right). E. Reduction in parasite burden is achieved when Th1-type immune responses become dominant. F. Resolution of infection is accompanied by granuloma involution (loss of cellularity) and a restoration of homeostasis. Experimental and modeling data suggest, however, that some residual parasites survive in some granulomas due to regulatory mechanisms.

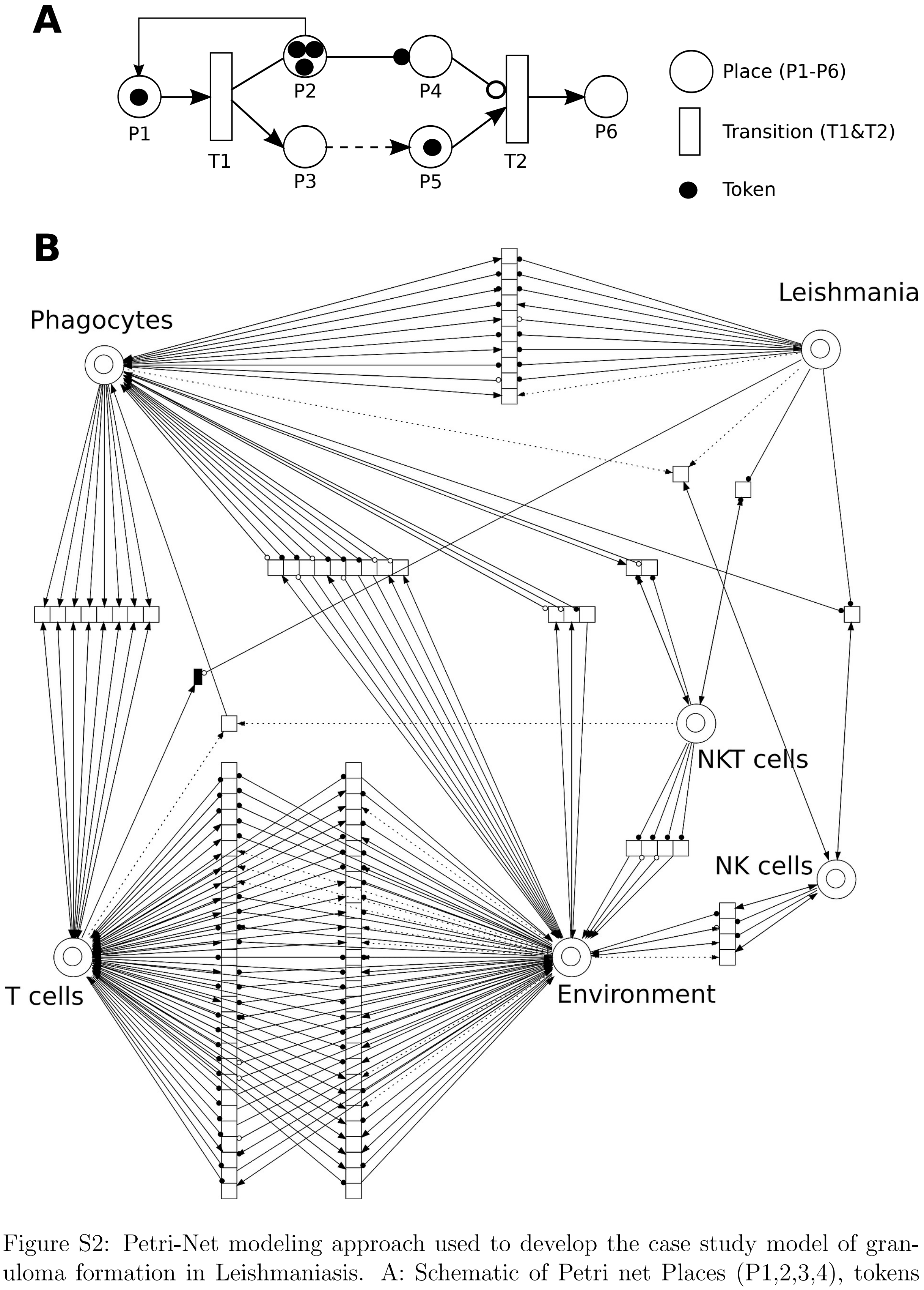

Figure S2: Petri-Net modeling approach used to develop the case study model of granuloma formation in Leishmaniasis. A: Schematic of Petri net Places (P1,2,3,4), tokens (black circles in places) and transitions (T1,2). Continuous line, Standard arrowhead: takes tokens from the input places and moves tokens to the output place. Dotted Line, Standard arrowhead: The number of tokens of a place is used in the evaluation of the rate of a transition. Continuous line, Full circle: Target transition only performed if the appropriate number of tokens is present in input. Continuous line, Empty circle: Disables the target transition if the appropriate number of tokens is present in the input place. B: High level Petri-Net model of granuloma formation, reproduced from [3].

Semantics Used in Artoo Argumentation Tool

In Context Of
Supported By

Notation	Definition	Description	Connected To
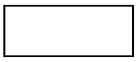	Claim	A Claim is an identified fitness-for-purpose requirement that the argument is seeking to substantiate, if possible. As an argument around a claim is constructed, that argument can be broken down into subsets of claims that, if substantiated, support the substantiation of the higher level claim	Context<br>Assumption<br>Strategy<br>Evidence
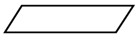	Strategy	A Strategy node should state the specific actions that have been taken to substantiate the claim to which this node is attached. This strategy may consist of breaking the claim down into a subset of claims which are then argued in turn.	Context<br>Assumption<br>Justification<br>Sub-Claim<br>Unsubstantiated Claim<br>Claim Continued
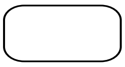	Context	A Context node should be used to provide contextual information concerning information in a node to which it is attached. This information may be definition of particular words or phrases (such as adequate) or the level at which the attached claim is deemed to have been substantiated.	
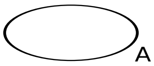	Assumption	An Assumption node provides a means of specifying any information that is assumed to be true when arguing over a claim or designing a strategy to examine a particular claim. Explicitly stating the inherent assumptions eases the process by which others can assess the extent to which the argument over a particular claim holds.	
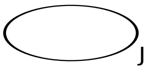	Justification	A Justification node should contain the reasoning for the application of a particular strategy in order to substantiate a claim. Justifying the approach used explicitly can reveal the extent to which alternative strategies have been considered, and why this strategy was selected over those alternatives.	
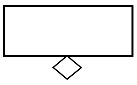	Unsubstantiated Claim	Unlike the application of structured notation in formal safety-case arguments, a claim can be shown to be unsubstantiated in the approach described in this paper. Biological systems are not fully understood, and it may not be possible to generate evidence to substantiate the complete set of fitness for purpose requirements. Where this is the case, it is critical that the lack of evidence is explicitly stated in the argument, and the limitations of the model are shown. In our approach, a lack of substantiating evidence is shown by attaching a white diamond to that claim	
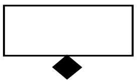	Claim Continued	As the argument becomes more complex, it may become difficult to follow. As such we have introduced a black diamond notation, representing the continuation of the argument surrounding this claim on a different diagram	
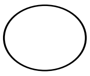	Evidence	Node containing the Evidence that is used to substantiate an attached claim. In Artoo, it is possible to hyperlink to this evidence, which could include publications, experimental results, statistical analyses, etc.	

Figure S3: Semantics of diagram language used in Artoo

Box 1: Developing a Claim

A Identify a **claim** that the argument seeks to support. This is represented within a rectangle. The objective is to detail how this claim can be supported with available evidence, if possible.

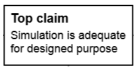

B Each **claim** is usually examined within a given **context**. This may involve defining the meaning of key terms stated in the claim. For example, if the purpose of the model was to adequately capture a biological system, adequate must be defined. Any context definitions are given in rounded rectangles

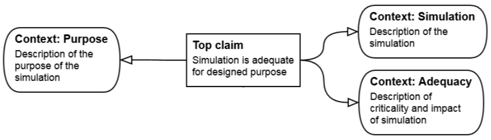

C Each **claim** is accompanied by one or more **strategies** that will be used to determine if that claim can be supported. This could, as examples, be a particular experimental strategy or systematic literature review. A strategy is always stated in a parallelogram.

A **claim** or **strategy** can also be accompanied by a **justification** or **assumption** node to provide more detail on the choice of the claim being made or the strategy that was followed. Semantics in Supplementary Figure 1

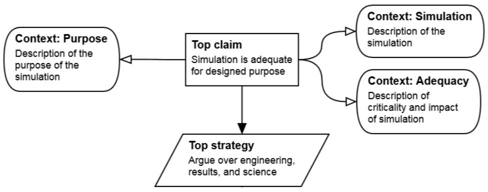

D **Strategies**, unless leading to **evidence** (see part E), are then broken down into **sub-claims**, and the process repeated. In this case, the strategy is divided into four sub-claims, each of which examines a key part of the model development process.

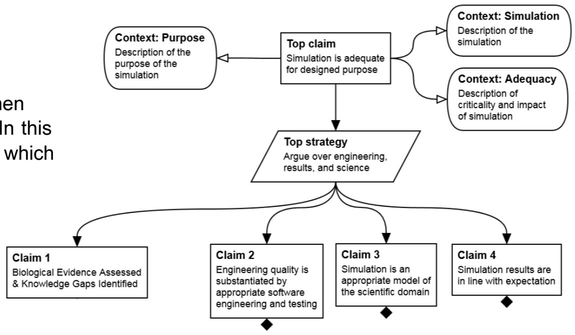

E If evidence can be provided that supports a claim, this is stated in an **evidence** node: a circle. In Artoo, electronic links to this evidence can be provided.
Diamonds on the diagram indicate either:
(i) a **claim cannot be supported**. If no evidence can be provided, a white diamond can be used to show this is the case.
(ii) the **argument is detailed on another diagram.** A black diamond is used to show the claim is fully described elsewhere.

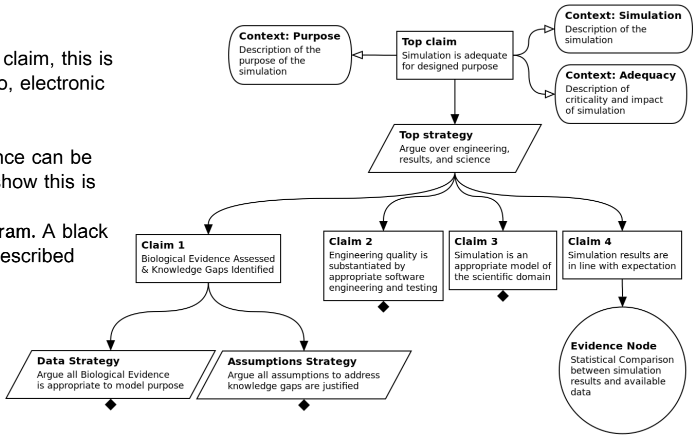

Figure S4: Process of developing a specific claim, using the diagrammatic notation used in Artoo.

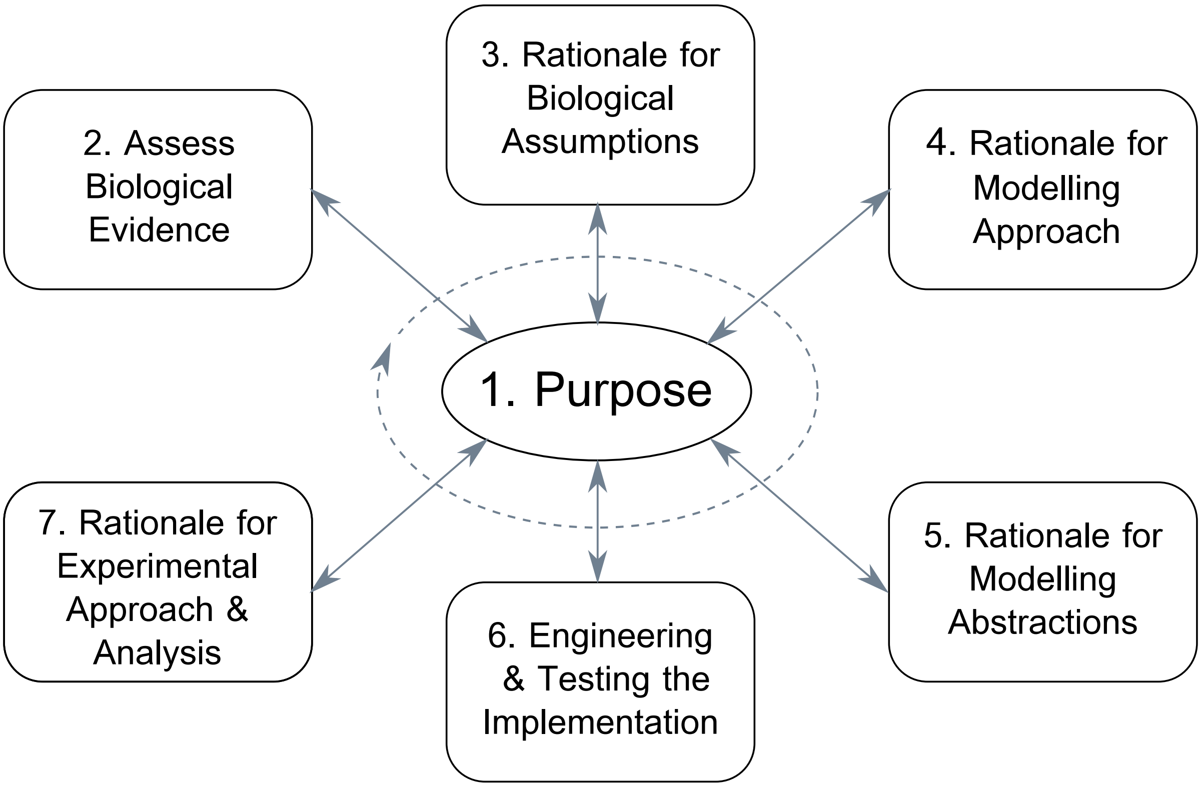

Figure S5: Process through which assessing the rationale for model design, implementation, and analysis should be conducted. Each stage of the process is grounded in the purpose for which the model was developed. Arrows linking to Purpose are bidirectional as the purpose shapes what assumptions and abstractions are appropriate, and conversely, decisions about assumptions and abstractions that are made can de facto alter the purpose for which the model is fit. Note the lack of defined end point: arguing fitness for purpose has potential to inform later iterations of model and study development.
