# Supplementary figures and images for: Building confidence in quantitative systems pharmacology models: An engineer's guide to exploring the rationale in model design and development
Source: CPT Pharmacometrics Syst Pharmacol. 2017 Feb 9;6(3):156–67. doi: 10.1002/psp4.12157 (PMC5351409; doi:10.1002/psp4.12157)

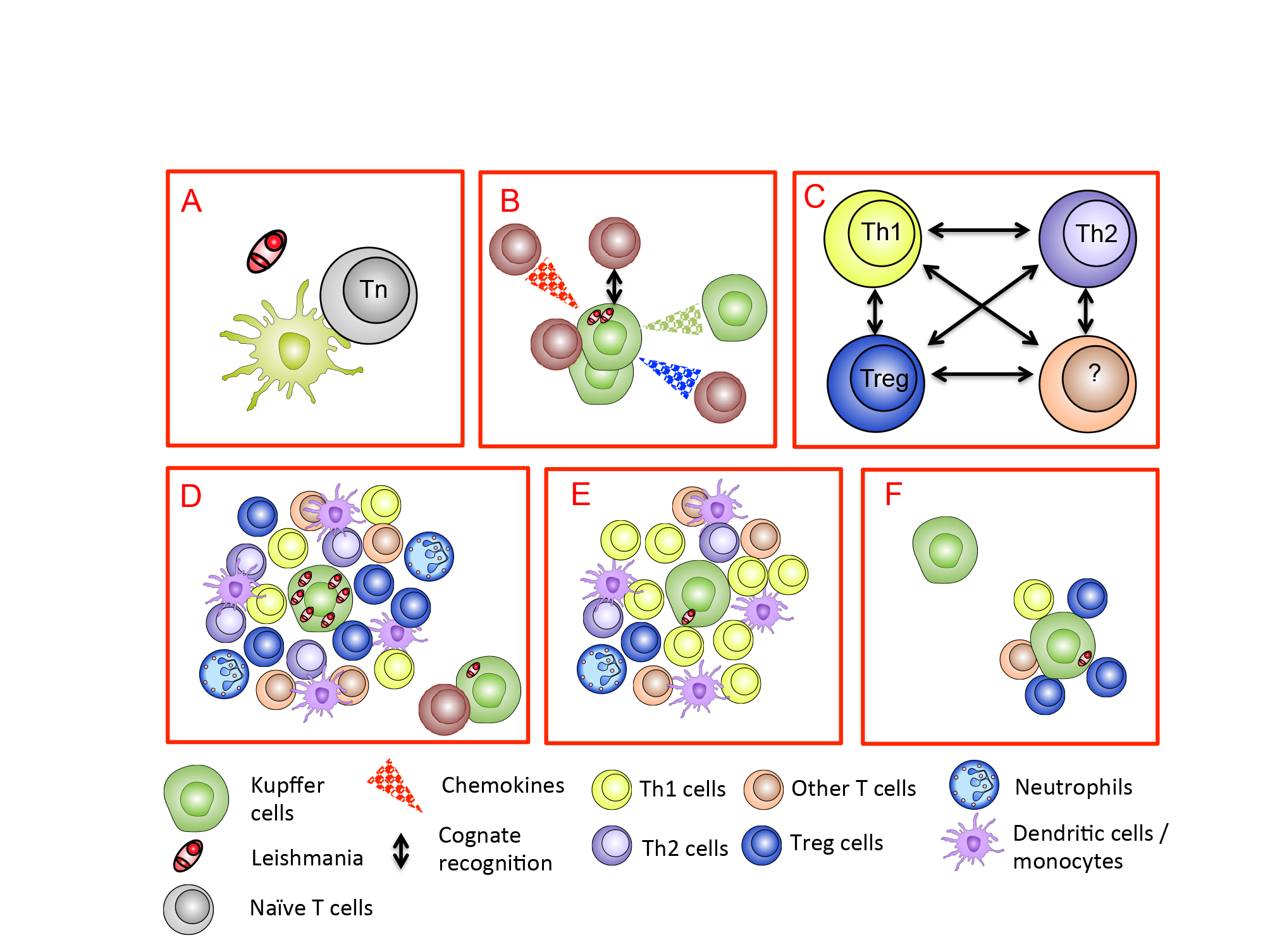

Supplement: Supplementary file 2 — Supplementary Figure S2 Petri net modeling approach used to develop the case study model of granuloma formation in Leishmaniasis. (a) Schematic of Petri net places (P1, 2, 3, and 4), tokens (black circles in places) and transitions (T1 and T2). Continuous line, standard arrowhead: takes tokens from the input places and moves tokens to the output place. Dotted line, standard arrowhead: the number of tokens of a place is used in the evaluation of the rate of a transition. Continuous line, full circle: target transition only performed if the appropriate number of tokens is present in input. Continuous line, empty circle: disables the target transition if the appropriate number of tokens is present in the input place. (b) High‐level Petri net model of granuloma formation, reproduced from Ref. 11. [file PSP4-6-156-s002.tif]

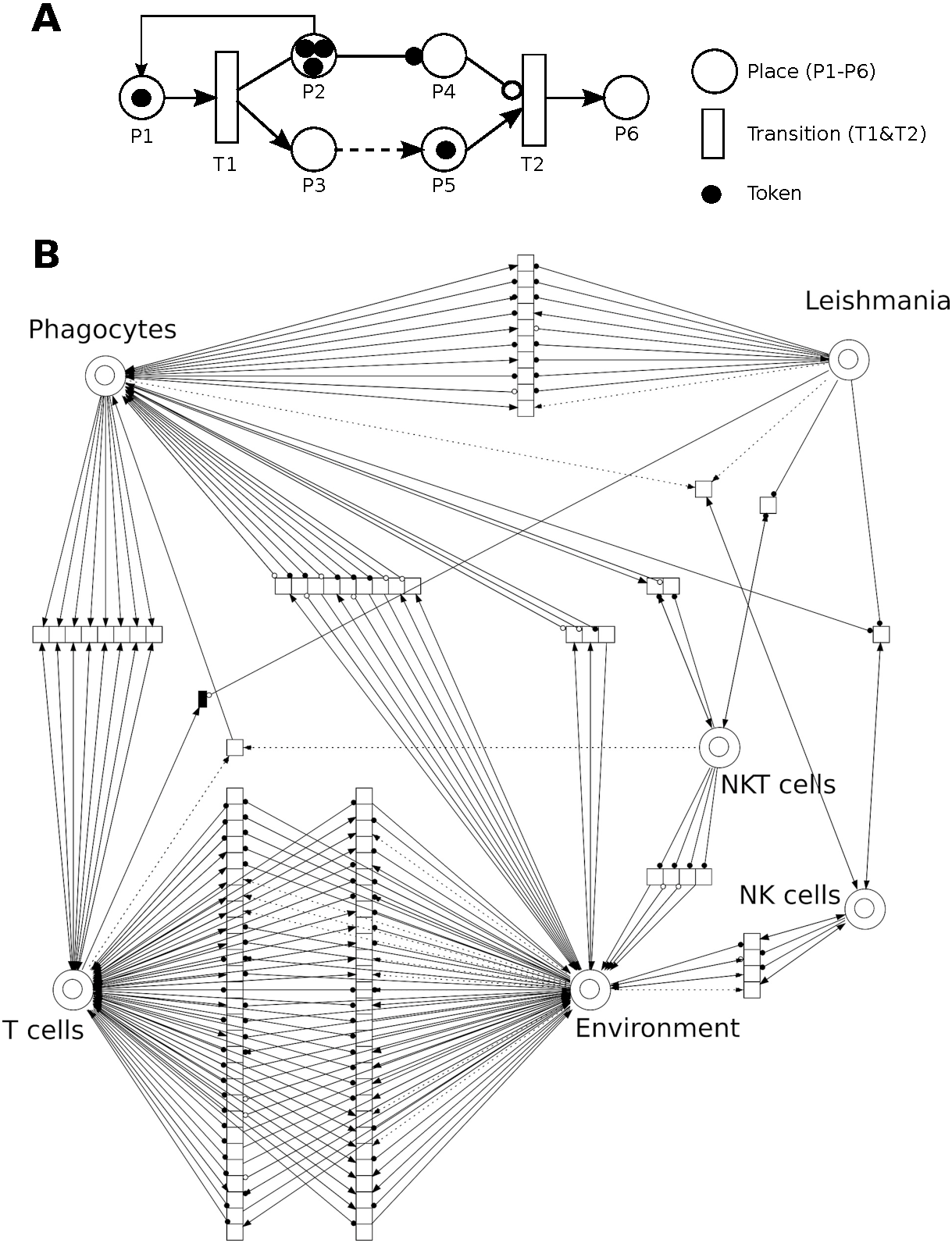

Supplement: Supplementary file 3 — Supplementary Figure S3 Semantics of diagram language used in Artoo. [file PSP4-6-156-s003.tif]

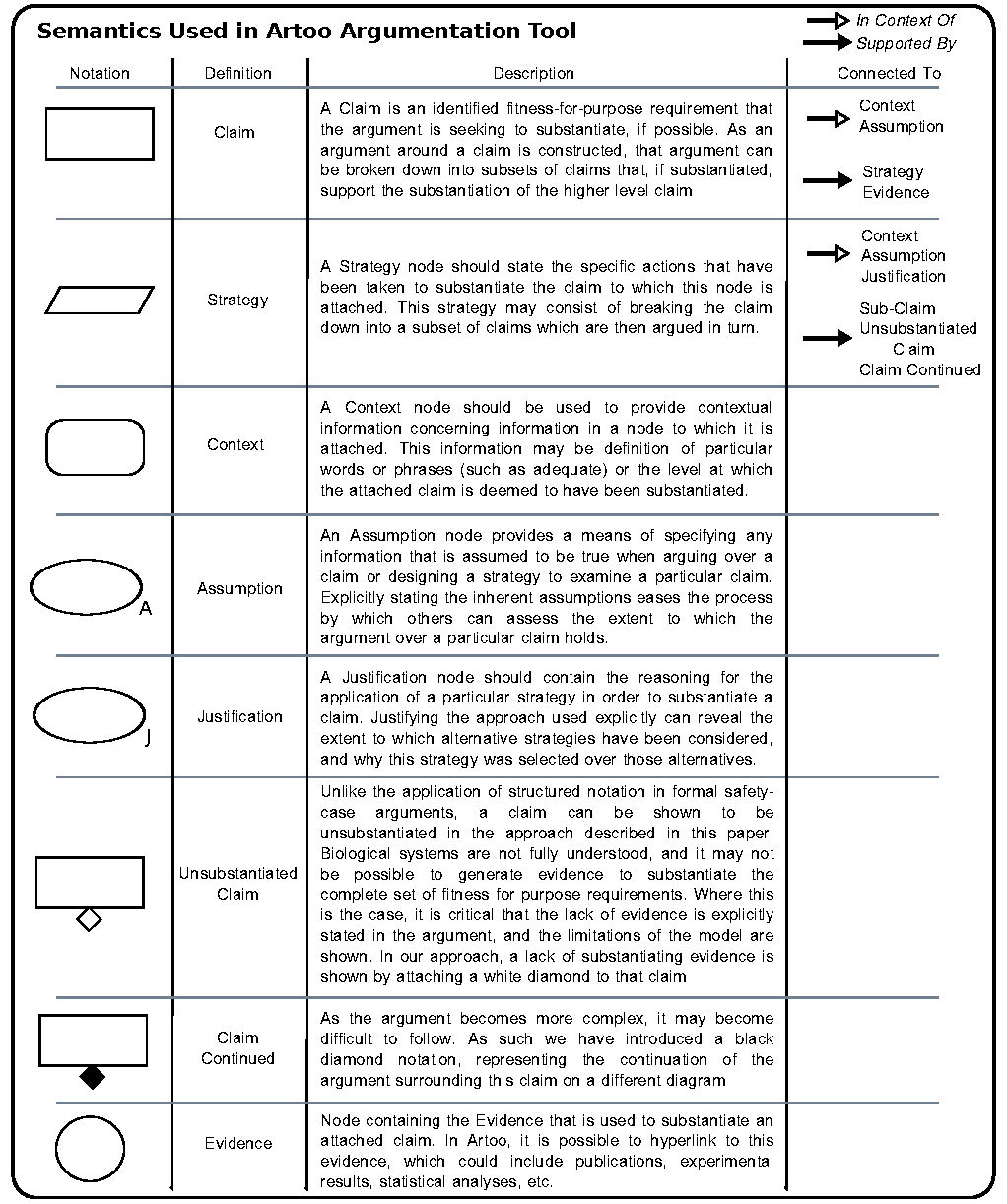

Supplement: Supplementary file 4 — Supplementary Figure S4 Process of developing a specific claim using the diagrammatic notation used in Artoo. [file PSP4-6-156-s004.tif]

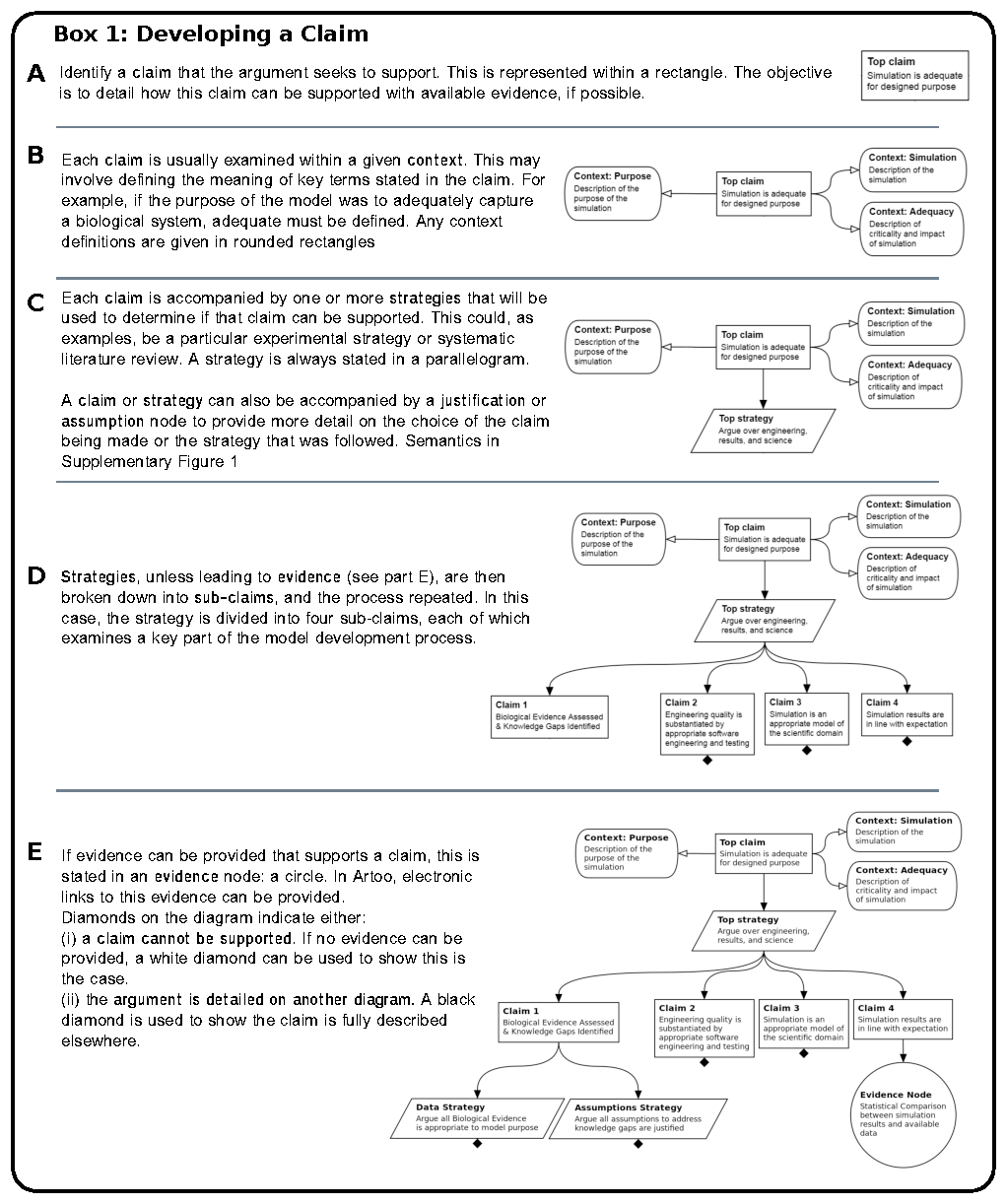

Supplement: Supplementary file 5 — Supplementary Figure S5 Process through which assessing the rationale for model design, implementation, and analysis should be conducted. Each stage of the process is grounded in the purpose for which the model was developed. Arrows linking to purpose are bidirectional as the purpose shapes what assumptions and abstractions are appropriate, and conversely, decisions about assumptions and abstractions that are made can de facto alter the purpose for which the model is fit. Note the lack of defined endpoint: arguing fitness for purpose has potential to inform later iterations of model and study development. [file PSP4-6-156-s005.tif]

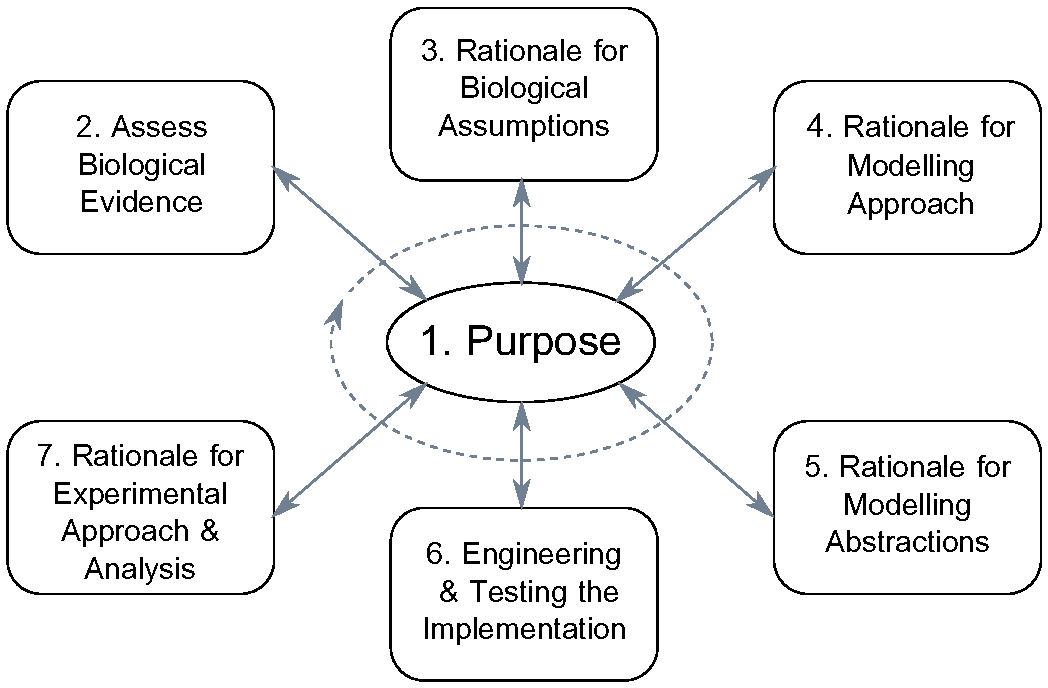

Supplement: Supplementary file 6 — Supporting Information S6 [file PSP4-6-156-s006.tif]
